# *Synallactesmcdanieli* sp. nov., a new species of sea cucumber from British Columbia, Canada and the Gulf of Alaska, USA (Holothuroidea, Synallactida)

**DOI:** 10.3897/BDJ.12.e124603

**Published:** 2024-06-26

**Authors:** Francisco A Solís Marín, Andrea A Caballero Ochoa, Carlos A Conejeros-Vargas

**Affiliations:** 1 Colección Nacional de Equinodermos “M. Elena Caso M.”, Laboratorio de Sistemática y Ecología de Equinodermos, Instituto de Ciencias del Mar y Limnolog, Mexico City, Mexico Colección Nacional de Equinodermos “M. Elena Caso M.”, Laboratorio de Sistemática y Ecología de Equinodermos, Instituto de Ciencias del Mar y Limnolog Mexico City Mexico; 2 Facultad de Ciencias, UNAM. Circuito exterior s/n, Ciudad de México, C. P. 04510, México., Ciudad de Mexico, Mexico Facultad de Ciencias, UNAM. Circuito exterior s/n, Ciudad de México, C. P. 04510, México. Ciudad de Mexico Mexico

**Keywords:** Synallactidae, taxonomy, Northeast Pacific

## Abstract

**Background:**

The family Synallactidae comprises mostly deep-sea forms and is the least-studied large taxon amongst deep-sea cucumbers. They are part of the abyssal megafauna and play an important role in modifying the sediment landscape and structuring the communities that live within it. The family embraces the genus *Synallactes*, which contains approximately twenty-five species from the Pacific, Atlantic (six species), Indian (seven species) and Antarctic Oceans (one species).

**New information:**

*Synallactesmcdanieli* sp. nov. is described from the Northeast Pacific, Knight Inlet, British Columbia, Canada to Kodiak Island, Gulf of Alaska, USA, at depths from 21 to 438 m. This new species is unique amongst the species of the genus *Synallactes* because of the number and arrangement of dorsal papillae, number of Polian vesicles, together with the entire ossicle arrangement. In addition, this species has the shallowest bathymetric distribution ever recorded for this genus.

## Introduction

The family Synallactidae Ludwig, 1894 comprises mostly deep-sea forms and is the least-studied large taxon amongst deep-sea cucumbers (Solis-Marin 2005). Synallactids are one of the most characteristic animals of the deep ocean. They often appear in photographic collections of abyssal megafauna (Bluhm and Gebruk 1999, Smet et al. 2021). Many of these photographs show their characteristic tracks and faecal remains (Young et al. 1985, Bluhm and Gebruk 1999, Bribiesca-Contreras et al. 2022) providing evidence of their important role in modifying the sediment landscape and in structuring the communities that live within it (Roberts et al. 2001). The majority of synallactids appear to spend their life on the sediment surface and some species are capable of active swimming, such as *Bathyplotesnatans* (Sars, 1868) and *Paelopatidesconfundens* Théel, 1886 (Miller and Pawson 1990). The epibenthic species traverse the seabed, feeding on the uppermost layer of sediment, for example, *Mesothuriaverrilli* (Theel, 1886).

The family Synallactidae formerly belonged in the order Aspidochirotida Grube, 1840, but was later transferred to the order Synallactida Miller, Kerr, Paulay, Reich, Wilson, Carvajal and Rouse, 2017. The order Synallactida includes the families Deimatidae Theel, 1882, Stichopodidae Haeckel, 1896 and Synallactidae Ludwig, 1894. The last family embraces the genus *Synallactes* Ludwig, 1894, which contains approximately twenty-five species. As far as we know, eleven of these species occur in the Pacific Ocean: *Synallactesaenigma*, *S.alexandri*, *S.chuni*, *S.discoidalis*, *S.gilberti*, *S.horridus*, *S.multivesiculatus*, *S.nozawai*, *S.sagamiensis*, *S.triradiata* and *S.virgulasolida*. The remaining species inhabit the Atlantic Ocean (six species), the Indian Ocean (seven species) and the Antarctic Ocean (one species). The purpose of this paper is to describe a new species of *Synallactes* from the Northeast Pacific.

## Materials and methods

Specimens are housed in the Royal British Columbia Museum, Invertebrate Zoology Collection, Victoria, B.C., Canada. Ossicles were extracted from the body wall (anterior, middle and posterior regions), dorsal papillae, ventral tube feet, tentacles and gonads. The tissue was dissolved in fresh household bleach (5–6.5%). After centrifugation at 1000 rpm for 10 min, bleach was pipetted off and the ossicles were rinsed and centrifuged with distilled water that was subsequently pipetted off. The same process was done with 70, 80 and 95% ethanol. Absolute ethanol was added to the ossicles and a small aliquot was placed to dry on a cylindrical double-coated conductive carbon tape stub. Then, it was sputter-coated with gold 2.5 kV in the ioniser JEOL JFC-1100 for 3 min and photographed using a JEOL JSM-6360LV scanning electron microscope (SEM) at the ICML, UNAM.

### Abbreviations used in the text:

**ICML**, UNAM Instituto de Ciencias del Mar y Limnología, Universidad Nacional Autónoma de México; **RBCM**, Royal British Columbia Museum, Victoria, British Columbia, Canada. **TL**, total length.

## Data resources

Specimens are housed in the Royal British Columbia Museum, Invertebrate Zoology Collection, Victoria, B.C., Canada.

## Taxon treatments

### Synallactes mcdanieli sp. nov.

56E8368E-C50E-5A00-A41D-8BB71A6C02B7

E9C929C1-ACEF-48BD-9646-281B376ED87D

*Synallacteschallengeri* - Edwards (1907): 65-66, text fig. 12; Lambert (1997): 39, 42-43, figs. 15-16, colour photo 4; Lamb and Hanby (2005): 340; Lambert and Boutillier (2011): 6 (list); Drumm et al. (2016): 261 (list).

#### Materials

- **Type status:** Holotype. **Occurrence:** catalogNumber: RBCM 995-00131-001; recordedBy: Philip Lambert; occurrenceID: 8130E68F-91C5-5657-8B36-28221E94FFBA; **Taxon:** taxonRemarks: 310 mm TL; **Location:** locality: West of Indian Cove near Auke Cape, Stephens Passage, Auke Bay, Alaska, USA.; verbatimDepth: 21 m; verbatimCoordinates: 58°22’29.95”N, 134°42’57.36”W; **Event:** eventDate: 13 July 1995
- **Type status:** Paratype. **Occurrence:** catalogNumber: RBCM 987-00380-020; recordedBy: Barry Boetter; individualCount: 1; occurrenceID: EBFBD103-E363-57DC-8E00-4E2092F9778B; **Taxon:** taxonRemarks: 225 mm TL; **Location:** locality: Portland Inlet, Alice Arm, British Columbia, Canada; verbatimDepth: 96 m; verbatimCoordinates: 55°24’46.85”N, 129°40’40.84”W; **Event:** eventDate: 27 October 1986
- **Type status:** Paratype. **Occurrence:** catalogNumber: RBCM 987-00381-009; recordedBy: Barry Boetter; individualCount: 3; occurrenceID: 258A7D85-A6F9-5CDE-AEA1-52A80032DEE5; **Taxon:** taxonRemarks: 205-267 mm TL; **Location:** locality: Portland Inlet; Alice Arm, British Columbia, Canada; verbatimDepth: 361 m; verbatimCoordinates: 55°27’01.13”N, 129°35’52.58”W; **Event:** eventDate: 26 October 1986
- **Type status:** Paratype. **Occurrence:** catalogNumber: RBCM 984-00259-001; recordedBy: Unknown; individualCount: 15; occurrenceID: 3FD8B5C0-7A05-5261-A466-5D697A62DF23; **Taxon:** taxonRemarks: 84-130 mm TL; **Location:** locality: SW of Baranof Island, Alaska, USA; verbatimDepth: 192 m; verbatimCoordinates: 56°17’59.88”N, 135°28’58.91”W; **Event:** eventDate: 3 March 1965
- **Type status:** Other material. **Occurrence:** catalogNumber: RBCM 988-00018-006; recordedBy: D. Graves and Rosenthal; individualCount: 1; occurrenceID: 84439A0A-A50B-51CC-81FA-166A1D7D779B; **Taxon:** taxonRemarks: 98 mm TL; **Location:** locality: Necker Bay, Baranof Island, Alaska, USA; verbatimDepth: 70 m; verbatimCoordinates: 56°36’17.79”N, 135°15’28.56”W; **Event:** eventDate: 10 August 1983
- **Type status:** Other material. **Occurrence:** catalogNumber: RBCM 984-00215-001; recordedBy: Frank Bernard; individualCount: 1; occurrenceID: 0E192BD6-23A5-5639-B30B-CCAD067792BE; **Taxon:** taxonRemarks: 55 mm TL; **Location:** locality: Dixon Entrance, British Columbia, Canada; verbatimDepth: 256 m; verbatimCoordinates: 54°28’33.45”N, 133°53’16.61”W; **Event:** eventDate: 19 September 1971
- **Type status:** Other material. **Occurrence:** catalogNumber: RBCM 984-00195-001; recordedBy: Dan Quayle; individualCount: 1; occurrenceID: 5E3CB5BB-9068-51B3-919F-360EE68088B3; **Taxon:** taxonRemarks: 87 mm TL; **Location:** locality: SE of Kodiak Island, Alaska, USA; verbatimDepth: 128 m; verbatimCoordinates: 56°42’28.74”N, 153°18’57.45”W; **Event:** eventDate: 15 September 1963
- **Type status:** Other material. **Occurrence:** catalogNumber: RBCM 983-01584-002; recordedBy: D. Graves; individualCount: 2; occurrenceID: 35AA21F7-CC61-59BC-80C4-8A376349FE12; **Taxon:** taxonRemarks: 110-115 mm TL; **Location:** locality: off Baranof Island, 15 miles NW of Larch Bay, Alaska, USA; verbatimDepth: 213-222 m; verbatimCoordinates: 56°13’12.00”N, 134°48’57.60”W; **Event:** eventDate: 8 August 1983
- **Type status:** Other material. **Occurrence:** catalogNumber: RBCM 983-01590-004; recordedBy: Alex Peden; individualCount: 1; occurrenceID: 9AA7ACD4-D440-551E-B1BE-E25D8FC47275; **Taxon:** taxonRemarks: 60 mm TL; **Location:** locality: off Salisbury Sound , Southwest of Chichagof Island, Alaska, USA; verbatimDepth: 170 m; verbatimCoordinates: 57°19’00.00”N, 136°00’57.51”W; **Event:** eventDate: 1 August 1983
- **Type status:** Other material. **Occurrence:** catalogNumber: RBCM 984-00110-001; recordedBy: Norm Sloan; individualCount: 1; occurrenceID: 44E906D0-E490-5AC5-86D2-C59A98323229; **Taxon:** taxonRemarks: 100 mm TL; **Location:** locality: south end of Observatory Inlet, Portland Inlet, British Columbia, Canada; verbatimDepth: 380 m; verbatimCoordinates: 55°07’56.96”N, 129°55’40.72”W; **Event:** eventDate: 1 November 1983
- **Type status:** Other material. **Occurrence:** catalogNumber: RBCM 984-00418-005; recordedBy: Alex Peden and Brent Cooke; individualCount: 1; occurrenceID: 8D0D0F85-4D5E-5A99-A79D-5F2996EC165B; **Taxon:** taxonRemarks: 170 mm TL; **Location:** locality: south shore, east of Tasu Narrows, Moresby Island, Haida Gwaii, British Columbia, Canada; verbatimDepth: 132 m; verbatimCoordinates: 52°45’05.59”N, 132°04’53.83”W; **Event:** eventDate: 15 September 1984
- **Type status:** Other material. **Occurrence:** catalogNumber: RBCM 984-00260-001; recordedBy: unknown; individualCount: 3; occurrenceID: DC6EB69A-7580-51EB-A832-B7BEDFDC4D25; **Taxon:** taxonRemarks: 79-99 mm TL; **Location:** locality: NW of Chichagof Island, Alaska, USA; verbatimDepth: 366 m; verbatimCoordinates: 57°51’59.55”N, 136°49’59.88”W; **Event:** eventDate: 28 August 1965
- **Type status:** Other material. **Occurrence:** catalogNumber: RBCM 976-01038-022; recordedBy: Philip Lambert; individualCount: 5; occurrenceID: DB4BBB4E-CDDC-5148-A0CA-4D5778F090DC; **Taxon:** taxonRemarks: 30-90 mm TL; **Location:** locality: north point of Belle Bay, opposite Hatie Island, Portland Canal, British Columbia, Canada; verbatimDepth: 22.9 m; verbatimCoordinates: 55°17’51.64”N, 129°57’35.57”W; **Event:** eventDate: 28 March 1976
- **Type status:** Other material. **Occurrence:** catalogNumber: RBCM 983-01588-002; recordedBy: Mermaid II dive 44, Alex Peden and Greg Brown; individualCount: 1; occurrenceID: C26CB4A7-C58C-5994-90A9-194E523DA503; **Taxon:** taxonRemarks: 80 mm TL; **Location:** locality: Salisbury Sound, SW of Chichagof Island, Alaska, USA; verbatimDepth: 77-80 m; verbatimCoordinates: 57°19’47.95”N, 136°00’59.85”W; **Event:** eventDate: 12 August 1983
- **Type status:** Other material. **Occurrence:** catalogNumber: RBCM 984-00256-002; recordedBy: Dan Quayle; individualCount: 6; occurrenceID: F9307681-8EDA-50AA-ABBA-BA8436BBB2E9; **Taxon:** taxonRemarks: 83-120 mm TL; **Location:** locality: west of Calvert Island, British Columbia, Canada; verbatimDepth: 247 m; verbatimCoordinates: 51°19’47.95”N, 129°04’59.88”W; **Event:** eventDate: 25 August 1965
- **Type status:** Other material. **Occurrence:** catalogNumber: RBCM 987-00379-012; recordedBy: Barry Boettger; individualCount: 2; occurrenceID: 35B4D6AE-E99A-58A2-9DFF-0EAEB3B8E380; **Taxon:** taxonRemarks: 165-170 mm TL; **Location:** locality: Portland Inlet, Alice Arm, British Columbia, Canada; verbatimDepth: 349 m; verbatimCoordinates: 55°26’45.56”N, 129°33’36.00”W; **Event:** eventDate: 26 October 1986
- **Type status:** Other material. **Occurrence:** catalogNumber: RBCM 973-00199-028; recordedBy: Alex Peden; individualCount: 1; occurrenceID: 6546057B-BB9E-53B8-9B6B-362C3C182DE2; **Taxon:** taxonRemarks: 54 mm TL; **Location:** locality: Queen Charlotte Sound, British Columbia, Canada; verbatimDepth: 46 m; verbatimCoordinates: 51°07’48.11”N, 129°26’11.58”W; **Event:** eventDate: 7 September 1973
- **Type status:** Other material. **Occurrence:** catalogNumber: RBCM 976-01080-016; recordedBy: Philip Lambert; individualCount: 1; occurrenceID: DF0AF0B3-AA7F-5690-B4A7-33573420A376; **Taxon:** taxonRemarks: 160 mm TL; **Location:** locality: Tasu Sound, off the small island south of Hunger Harbour, British Columbia, Canada; verbatimDepth: 27 m; verbatimCoordinates: 52°45’17.96”N, 132°00’54.00”W; **Event:** eventDate: 20 August 1976

#### Description

**Holotype description.** Specimen 310 mm long; firm, slightly rough skin. Colour in alcohol light violet, the dorsal side more colourful than the ventral area where the prevailing colour is whitish-beige. Body subcylindrical, slightly flattened, more tapering posteriorly than anteriorly. Mouth ventral, anus terminal, both surrounded by small papillae (1.0-1.7 mm long). Peltate tentacles 20, each with 9-10 distal digitations. Subcylindrical tube feet ventrally (0.8-3 mm long), restricted to the ventral ambulacra. Distal end of feet with supporting sucking discs. The odd ambulacrum has two zigzag rows of about 62 tube feet each, ventrolateral ambulacra each with a zigzag row of 45 tube feet along the margin of ventral side. On the dorsal side are long papillae, 13 mm long and 4 mm across at base, most situated on conical warts. They form four parallel rows, each consisting of about 25-30 papillae. Papillae of the central dorsum are larger than those of the rest. Much smaller papillae belonging to ventrolateral ambulacra form a marginal fringe around the mouth and anus.

**Figure 1.**
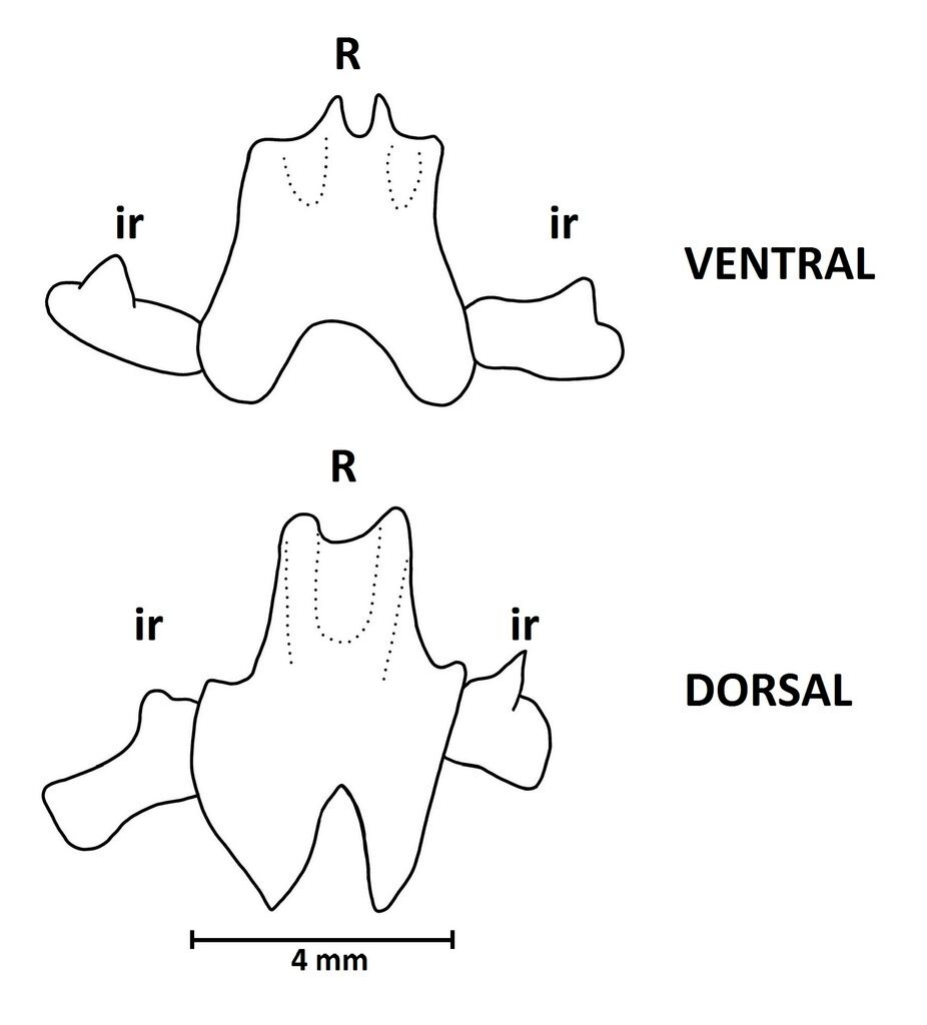
*Synallactesmcdanieli* sp. nov. Holotype RBCM 995-00131-001. Calcareous ring. Single ventral and dorsal radials (**R**) and adjoining interradial plates (**ir**).

Calcareous ring composed of five radial and five interradial plates. Small interradial pieces with one central anterior process (Fig. 1) and massive radial pieces with a posterior notch. The stone canal is fixed dorsally to the skin by the madreporic plate. There is one Polian vesicle. There are two well-developed respiratory trees, branched, occupying almost the entire length of the body. They consist of a long common stem which bifurcates into two short vessels. Gonad branched, disposed in two tufts. The longitudinal muscles are not divided.

*Ossicles*. There are few ossicles in the dorsal and ventral skin. Most are in the dorsal papillae, the ventral tube feet and the tentacles. Internally there are very scarce ossicles in the gonads.

**Figure 2.**
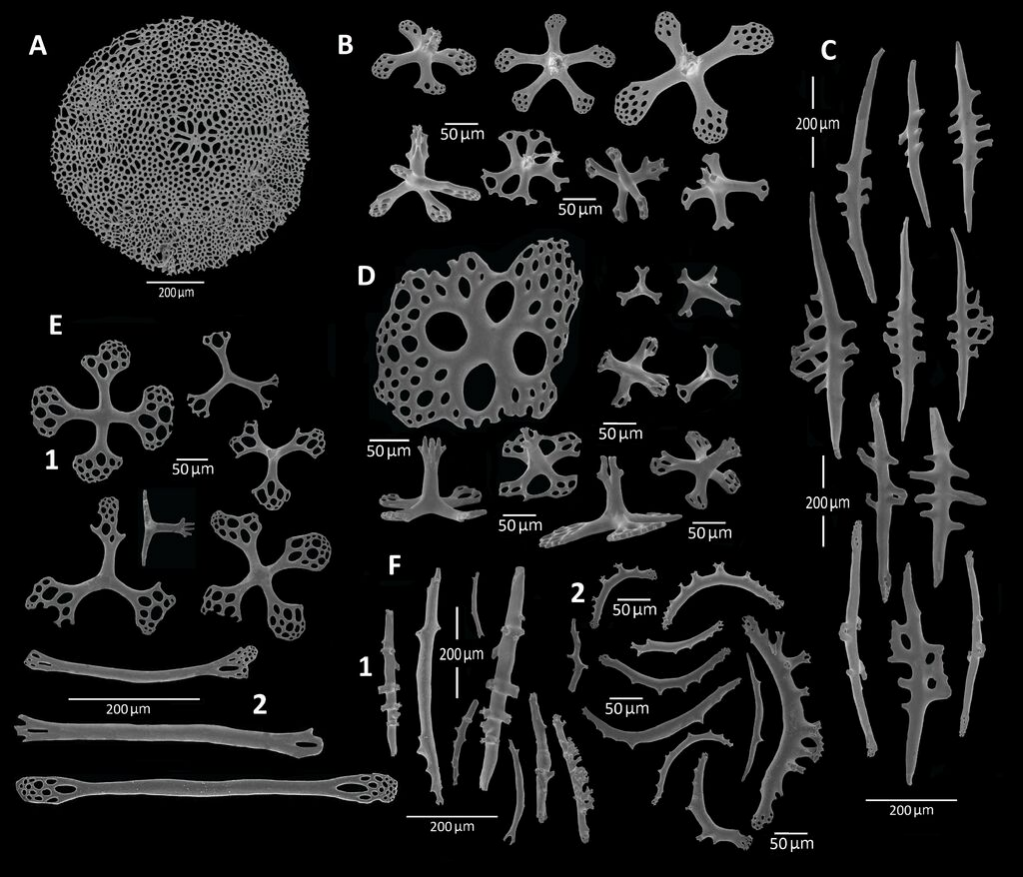
*Synallactesmcdanieli* sp. nov. Holotype RBCM 995-00131-001. Ossicles of tube feet (**A-C**) **A** Terminal plate; **B** quadri- and pentaradiate tables; **C** straight or curved rods. Ossicles of body wall (**D-E**) **D** tri- or quadri-radiate tables of ventral body wall; **E** Ossicles of dorsal body wall; **E1** tri- or quadri-radiate tables; **E2** straight rods; **F** Ossicles of the tentacles; **F1** straight, spiny rods; **F2** curved, forked, branched rods.

The body wall contains small (40-100 µm in diameter) and large (250-316 µm in diameter) tri- or quadri-radiate tables (Fig. 2D and E1). The end of each arm is bifurcated several times or perforated and spatulate in shape, some of them forming a brief lattice-like network. Centrally, there is one pillar (50-60 mm tall in the small tables and 70-100 mm tall in the large tables) which may be terminally divided in a single point or in 2-4 spines or perforated.

The tube feet contain rods (Fig. 2C and 2E2), quadri- and pentaradiate tables (Fig. 2B) and an end plate (Fig. 2A). The rods, which are straight or curved and sometimes forked, have perforated ends. They are 300-690 mm long. These rods are spiny, the lateral spines sometimes branched (Fig. 2C). The end plate reaches 1 mm in diameter and is composed of a single perforated plate (Fig. 2A).

**Figure 3.**
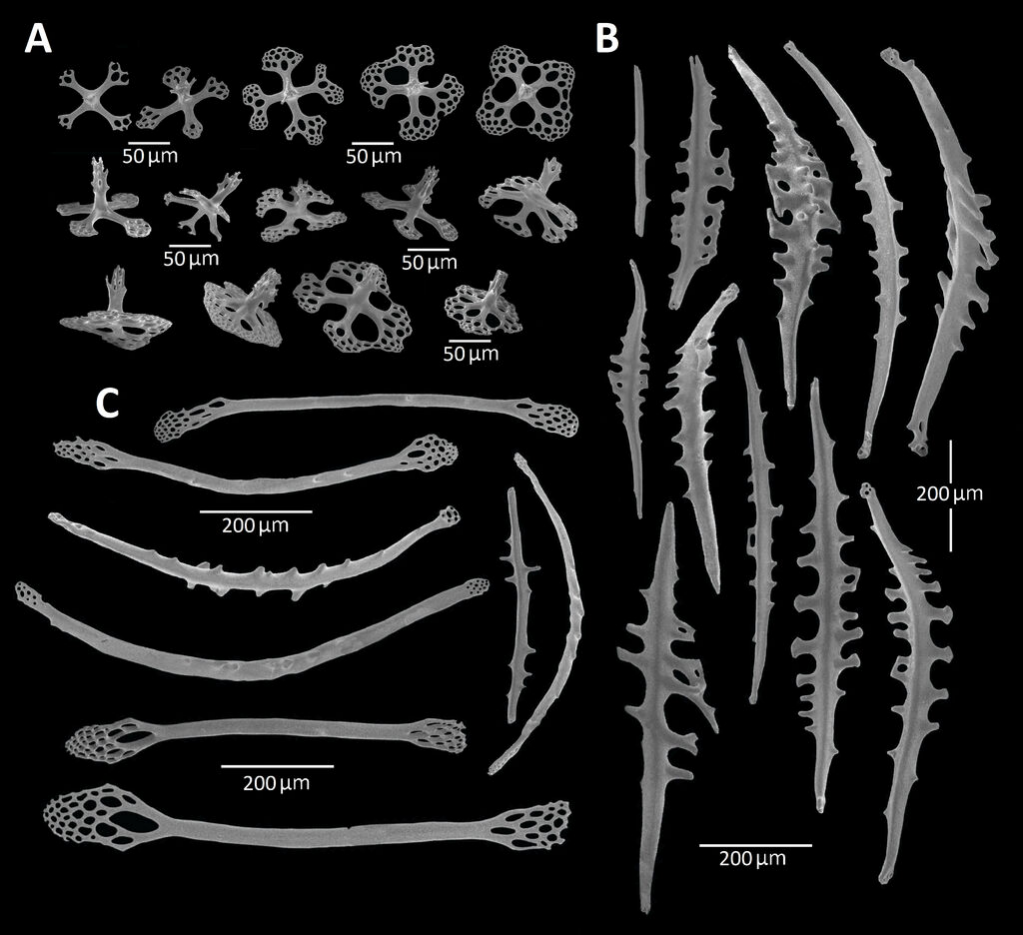
*Synallactesmcdanieli* sp. nov. Holotype RBCM 995-00131-001. Ossicles of dorsal papillae **A** tri-, quadri- and pentaradiate tables; **B** spiny rods, with the lateral spines (sometimes branched); **C** long, thin, smooth, straight or curved rods with perforated ends.

The dorsal papillae contain rods and tri-, quadri- and pentaradiate tables which are particularly densely packed at the tip of the papillae (Fig. 3A), some of them forming a brief lattice-like network. The rods at the base of the papillae (Fig. 3B and 3C) are similar to those of the tube feet, whereas the rods at the tip of the papillae are long (700-900 mm), thin and smooth with perforated ends (Fig. 3C). The quadri-radiate tables (Fig. 3A) are numerous and smaller (120-130 mm in diameter) than in the body wall.

**Figure 4.**
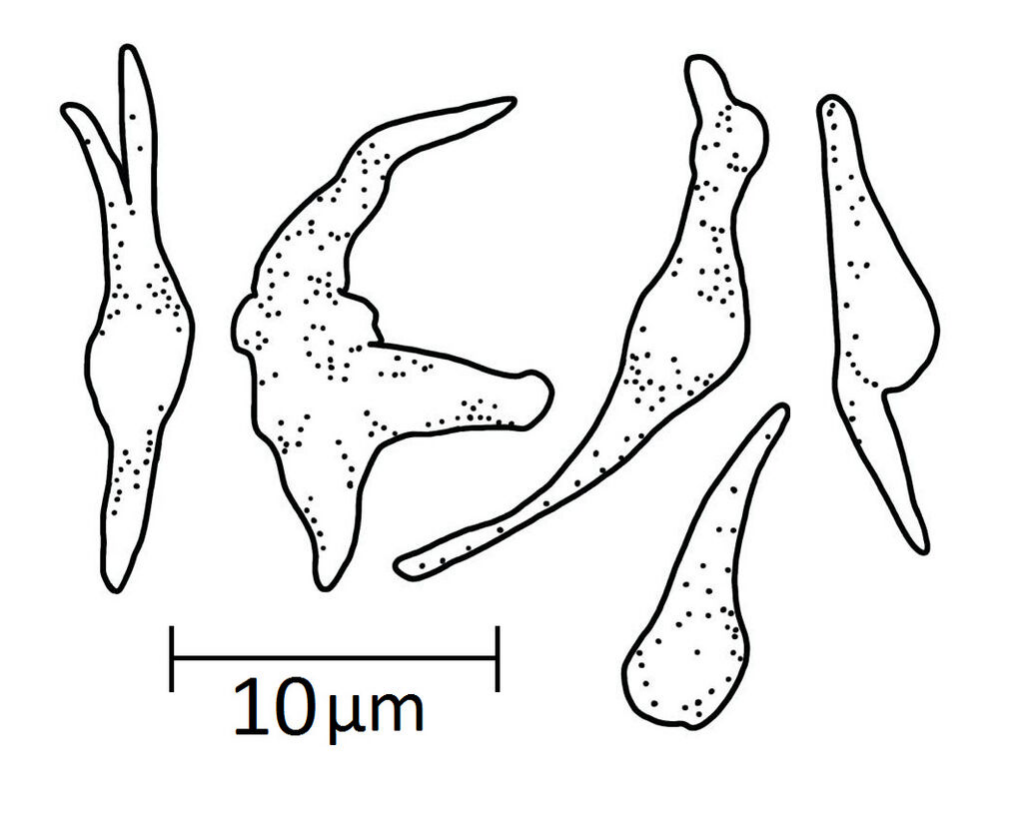
*Synallactesmcdanieli* sp. nov. Holotype RBCM 995-00131-001. Irregular calcareous bodies of the gonads.

The tentacles contain only rods which are straight (Fig. 2F1) or curved, forked and sometimes branched (Fig. 2F2). They are spiny and measure 400-700 mm long. Gonads with irregular calcareous bodies 10-20 mm (Fig. 4) branched and unbranched rods with pointed ends. Some rods with a single knobbed centre. Respiratory trees devoid of any ossicles.

**Figure 5.**
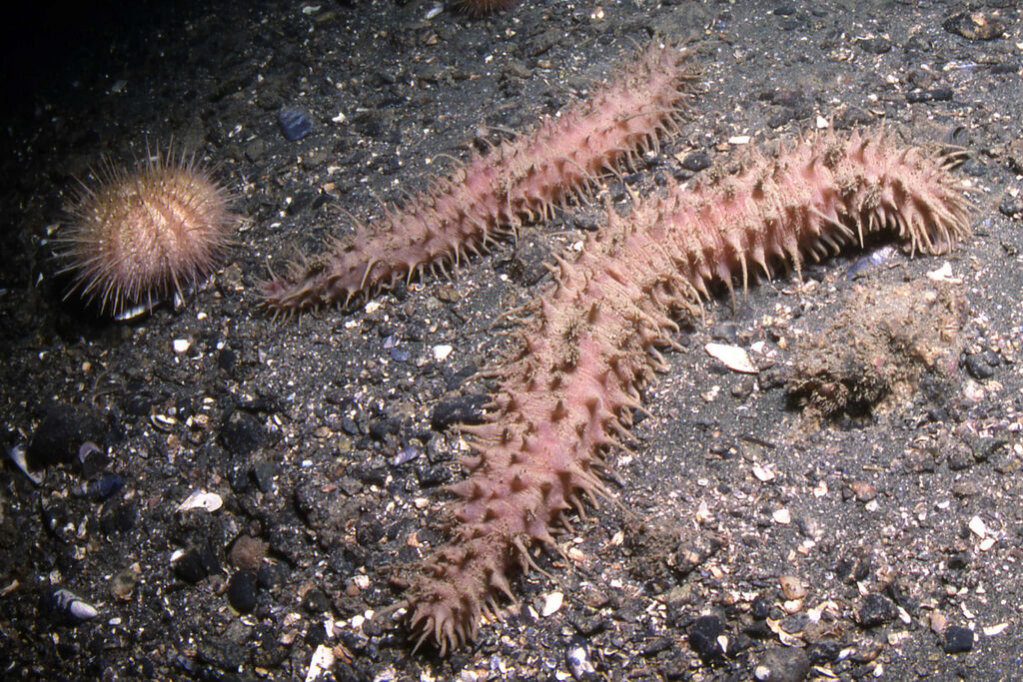
*Synallactesmcdanieli* sp. nov. In situ specimens at Battery Point, near Haines Alaska, USA, photo by Neil McDaniel. Approximately TL of specimens ~ 250-300 mm.

Colour of live specimens is pale pink violet on the dorsum and same colour, but lighter on the ventrum (Fig. 5). Preserved specimens can retain some violet colour on the dorsal area, but normally they are completely beige.

##### Paratype variations

Specimens range from 84-130 mm in length.

##### Type locality

West of Indian Cove near Auke Cape, Stephens Passage, Auke Bay, Alaska, USA 58°22’29.95”N, 134°42’57.36”W.

#### Diagnosis

Body subcylindrical, slightly flattened ventrally. Body wall slightly rough. Mouth ventral, anus terminal. Twenty peltate tentacles arranged in two concentric circles. Tube feet restricted to ventral ambulacra, short, cylindrical, each terminal adhesive disc possessing a large, perforated end plate. Two longitudinal series of tube feet along the latero-ventral radii and two longitudinal series in the mid-ventral radius. The tube feet are densely packed near the anus. Dorsal surface with conical papillae arranged in four longitudinal parallel rows at almost regular intervals. Ventro-lateral radii with long papillae. At the anterior end, papillae are longer than elsewhere. Calcareous ring well developed. Radial plates vary in robustness and shape depending on their position in the calcareous ring. Interradial plates almost of similar aspect and size. Polian vesicle single. Gonad branched, disposed in two tufts.

Ossicles: The body wall contains abundant tri-, quadri- or pentaradiate tables, with spatulated arm ends. The end of each arm is bifurcated several times or perforated, sometimes there are lateral processes which may unite some arms. The spire consists of a single pillar, which may be divided or perforated, or both, at the terminal end. One or two pairs of small, short, and robust spines project on the lateral sides of the upper end of the spire. There are tables, robust supporting spiny rods, and terminal disks in the tube feet. Papillae contain massive rods (smooth or branched), delicate rods and tables which are particularly densely packed at the tip of the papillae. Tri-, quadri- and pentaradiate tables are present. Tentacles with curved or straight spiny rods. Gonads with irregular calcareous bodies. Respiratory trees devoid of any ossicles.

#### Etymology

This species is named after Neil McDaniel, long-time Canadian marine naturalist, photographer and videographer, in recognition of his many contributions to marine sciences. The epithet is a noun in the genitive case.

#### Distribution

From Kodiak Island, Gulf of Alaska, USA to British Columbia, Canada. The southernmost distribution point is Hoeya Head, Knight Inlet, B.C., Canada (50°40’02.68”N, 126°00’28.04”W) as mentioned by Hakai Institute (Calvert Island, B.C.) dive group (Prentice et al. 2023) at 25 m deep on 12 October 2022, when they recorded the presence of “*Synallacteschallengeri*”.

#### Ecology

*Synallactesmcdanieli* sp. nov. was collected at 18 different stations between 21 and 380 m depth. Edwards (1907) mentions that the maximum depth of the specimens obtained in Alaska (as *S.challengeri*) was 438 m. The species occurs mainly on sandy-gravelly bottoms and amongst boulders and cobble substratum (Fig. 5). This species feeds on bottom sediments with its peltate tentacles like its congeners.

#### Taxon discussion

*Synallactesmcdanieli* sp. nov. shows affinities with the two *Synallactes* known from the Northeast Pacific, i.e. *S.nozawai* Mitsukuri, 1912 and *S.triradiata* Mitsukuri, 1912.

*S.nozawai* possesses an external morphology very similar to *S.mcdanieli* sp. nov., but differs in the number of dorsal papillae, six and four rows, respectively. Furthermore, ossicles in *S.nozawai* are nearly all quadri-radiate tables, very rarely tri-radiate, while in *S.mcdanieli* sp. nov., the body wall contains tri- and quadri-radiate tables. The table spires of *S.nozawai* can have from one to three holes at the tip and more spinelets at the top than those in *S.mcdanieli* sp. nov.

In addition to the more northern geographical distribution of *S.nozawai* (Bering Strait) in the Northeast Pacific, its bathymetric range (108-787 m) is deeper than in *S.mcdanieli* sp. nov. as currently known.

*Synallactestriradiata* is also very similar in external appearance to *S.mcdanieli* sp. nov., but has six longitudinal rows of dorsal papillae instead of four. Internally, *S.triradiata* differs from *S.mcdanieli* sp. nov. in having a variable number of polian vesicles (1-3) and the calcareous deposits are tri-radiate tables (arms of which stand 120˚ apart) with the spire terminating in several points. In addition to the above characteristics, *S.triradiata* inhabits Sagami Bay and Sagami Sea (Mitsukuri 1912) and the Northeast Pacific Ocean: Bering Sea, Alaska, Aleutian Islands, Fox Islands, Unalaska Bay, at depths from 640-1092 m (Solis-Marin 2003).

Edwards (1907) mentioned the existence of *S.challengeri* in the Gulf of Alaska based on six specimens collected between 87 to 438 m depth, on green mud, fine sand; this was followed by Lambert (1997) and Lambert and Boutillier (2011). As stated by Solis-Marin (2003) and Massin and Hendrickx (2010), the presence of *S.challengeri* along the west coast of North America up to the coast of California was putative and was in need of review. Indeed, *S.challengeri* is known from sub-Antarctic islands (HMS *Challenger* St. 148a, 46º 53’ S, 51º 52’ E, 990 m depth) (Théel 1886, Massin 1992); thus, we consider the specimens described by Edwards (1907) and Lambert (1997) to be *Synallactesmcdanieli* sp. nov. and not *S.challengeri*.

*Synallacteschallengeri* has a total length that varies from 69 to 160 mm (Théel 1886, Massin 1992, Thandar and Rambaran 2015), whereas *S.mcdanieli* sp. nov. is larger, ranging from 84 to 310 mm. As Théel mentioned in 1886, *S.challengeri* specimens have dispersed papillae on the ambulacral and interambulacral areas with visible rows laterally, while *S.mcdanieli* sp. nov. has dorsal conical papillae arranged in four longitudinal series confined to ambulacra at almost regular intervals. The number of Polian vesicles is also remarkable: *S.mcdanieli* sp. nov. has only one vesicle and, in *S.challengeri*, that number is variable from two to five (Théel 1886, Massin 1992). Quadri-radiate ossicles from the papillae vary from 20-350 mm in *S.challengeri*, while table sizes of *S.mcdanieli* sp. nov. are only large, from 300-320 mm. The new species has tri-, quadri- and pentaradiate tables densely packed at the tip the papillae and also massive (smooth or branched), delicate rods. The body wall of the two species has tri- and quadri-radiate tables with similar disc diameter (35-100 µm) and spire 40-80 µm tall. The main difference between the two species is that *S.mcdanieli* sp. nov. also has larger ossicles from 250-280 µm in diameter and a central pillar with a spire from 50-60 mm tall in the small tables and 70-100 mm tall in the big tables.

Species of the genus *Synallactes* are mostly found in deep water. Only three previously described species have their shallow bathymetric distribution limits at depths less than 200 m: *S.multivesiculatus* (194 m), *S.sagamiensis* (180 m) and *S.nozawai* (108 m). Only *S.mcdanieli* sp. nov. ranges from shallow (21 m) to deep water (438 m).

*Synallactesmcdanieli* sp. nov. is unique amongst the species of the genus *Synallactes* because of the number and arrangement of dorsal papillae and polian vesicles, together with the entire ossicle arrangement.

## Supplementary Material

XML Treatment for Synallactes mcdanieli
